# Different post-training processes in children's and adults' motor skill learning

**DOI:** 10.1371/journal.pone.0210658

**Published:** 2019-01-10

**Authors:** Esther Adi-Japha, Roni Berke, Nehama Shaya, Mona S. Julius

**Affiliations:** 1 School of Education, Bar-Ilan University, Ramat-Gan, Israel; 2 Gonda (Goldschmied) Multidisciplinary Brain Research Center, Bar-Ilan University, Ramat-Gan, Israel; 3 Special Education Studies, Levinsky College of Education, Tel Aviv, Israel; Waseda University, JAPAN

## Abstract

Do young children and adults share similar underlying motor skill learning mechanisms? Past studies have shown that school-aged children's speed of performance developed over wake periods of a few hours post-training. Such training-dependent gains were not found in adults. In the current study of children as young as 5-years-old and young adults who practiced a simple grapho-motor task, this finding was replicated only by the children that showed faster performance a few hours post-training. These positive gains in performance speed were retained two weeks later. Furthermore, among the children, variations in gains attained a few hours post-training were associated with initial performance level. These behavioral findings indicate different underlying post-training processes in children's and adults' motor skill learning thus, supporting differential tutoring of skills.

## Introduction

Skill learning is a basic mechanism, integral to the learning of cognitive, perceptual, motor, and linguistic skills, enabling newly acquired skills to improve gradually across multiple learning experiences [1]. Motor skill learning paradigms have been extensively used for studying the course and the underlying processes, of skill acquisition [2]. Motor skill learning initially develops relatively fast across different experimental paradigms (i.e., over the course of a single training session) and later slows as further gains develop incrementally over multiple practice sessions [3]. The progression from fast to slow motor skill learning is thought to depend on appropriate stabilization and consolidation processes [4, 5]. It has been shown in adults, school-aged children, and more recently in kindergarten children, that significant training-dependent gain in performance following a training experience can appear 24 hours post-training, presumably reflecting consolidation processes [6–8]. Following these and similar studies (for a review see, [9]), consolidation processes have been suggested as a means of explaining the benefit of distributing practice sessions across days (e.g., with a 24-hour between-session interval), for better performance and long-term retention of an acquired skill [10–12].

Recent studies conducted in children in multiple skill learning paradigms have shown that practice dependent gains may develop in a time interval of 1–12 hours in the awake state [13–17]. Two of the studies focused on a few hours' interval: Ashtamker and Karni [14], who studied the 5-item explicit finger-to-thumb opposition sequence in 10-year-olds, and Desrochers et al., [16] who studied the implicit serial reaction time task (SRTT) using a 5-item sequence in 3.5- to 6-year-olds. Note that in explicit paradigms, the individual is aware of acquiring the skill; however, this is not the case in implicit paradigms [5] The aforementioned studies facilitate the notion of early enhancement in children's motor learning, and challenge the notion of a 24-hour interval (or longer) to allow effective consolidation to take place. However, only studies that find a childhood advantage, when comparing the post-training gains of children with that of young adults over wake periods, can be taken as evidence for differential, age-dependent, post-training processes, and support differential tutoring of skills in children.

Only a few studies have compared the post-training performance of children with that of young adults. Studies conducted using explicit motor tasks ([17] and [14], which studied the 5-item explicit finger-to-thumb opposition sequence) suggested that the rate of development of the performance gains across wake periods was faster in children than in adults. Furthermore, in implicit motor tasks the performance of adults deteriorated during wakefulness, while that of children was maintained ([18]; The study examined the probabilistic SRTT in 7- to 11-year-olds and adults). The above reported studies, which underscore the children's advantage in post-training wakefulness processes, were conducted on school-aged children versus adults, and only one study [14] compared early gains. It is not clear if the earlier expression of enhancement also appears in younger children, relative to young adults, and in paradigms different from the 5-item sequence tapping tasks. Furthermore, it is not clear if early post-training gains are associated with the initial level of performance, as are training gains [8, 19]. It is also not clear if these gains are transient or stable across longer (weeks) retention intervals, because previous studies limited the report to 24 h post-training. This question is of interest because cellular [20–23] and neuroimaging studies [24] suggest that activity at short time scales (from a few minutes to a few hours after practice) is different than that observed at longer time scales (from days to weeks).

Several studies have examined the development of motor skill learning across childhood and into adulthood. Many of these studies point to a difference in skill learning between early and later childhood [25–29]. For example, on the mirror-tracing task [30], children younger than 7 years were not able to trace even one complete side of a diamond or a square correctly while looking at their hand only as a reflection in a mirror. Only children older than 10 years showed an adult-like pattern of learning [25, 29, 31]. However, prism adaption studies of juvenile and adult barn owls suggest that at least at the synaptic level, major changes occur at the time of sexual maturity and not before [23]. Differences have also been found in the role that sleep plays in memory consolidation in children. Prehn-Kristensen et al. [13] found that in 10- to 13-year-old children, sleep enhanced declarative emotional stimuli but not procedural memory; the latter was assessed using a mirror drawing task. Sleep may contribute in an indirect manner to the automation of motor adaptation skills as shown by increased proactive interference only following sleep in 10- to 12-year-old children [32].

One of the difficulties in testing motor skill learning in young children is the lack of age-appropriate tasks. Commonly used tasks, such as mirror tracing or the finger-to-thumb opposition sequence, were developed for adults, and young children struggle in performing these tasks [15, 17]. This may hinder the development of effective learning and memory assessments in children. In a recent series of studies, a writing-like "invented letter" task (ILT) appropriate for kindergarten children was introduced. In the ILT, participants connect three dots to form an invented letter [33]. It was shown that the ILT is suitable for use with 5- and 7-year-olds who showed similar learning phases to adults who were trained on the task [8]. The task was found to be predictive of these children's handwriting and reading a year later [29]. In the current study, training on the ILT and performance 2 hours or 4 hours post-training were studied in kindergarten children and in young adults, in order to test whether the children would show early expression of delayed gains. It should be noted that stabilization against interference in adults is achieved by 4 hours post-training, while children require less time for stabilizing performance [6, 34]. Late retention 2 weeks post-training was studied as well, in order to test whether early gains are retained and whether later gains are expressed in any of the groups.

Research Hypotheses

- Children will show positive gains of a similar magnitude of 2- and 4-hours post-training, while in adults performance level will be maintained at the end-of-training performance level.
- Children will maintain their level of 2/4 h post-training performance two weeks later, while in adults a significant improvement corresponding to consolidation phase gains is expected.

## Methods

The Ministry of Education (287/8918/2015) and the Bar-Ilan University School of Education Ethics Committee approved the study. All parents signed a Ministry of Education informed consent form. Adults signed a university-standard consent form.

### Participants

Forty-five kindergarten children (*M* = 71 months, SD = 2.45 months, range 65–79 months; 25 girls) and 40 university students (*M* = 28.23 years, SD = 5.5 years, range 19–37 years; 21 females) took part in the current study. The participants were recruited from centrally located areas with a medium to high socioeconomic status. All participants used their right hand to write or draw, and were right handed (children—kindergarten teacher report, adults—self report). All children could copy at least the first 8 figures of the Beery visual-motor integration test, indicating readiness for formal instruction in handwriting, adequate for performing the task [35, 8]

### Tasks

#### ILT

**Fig 1:**
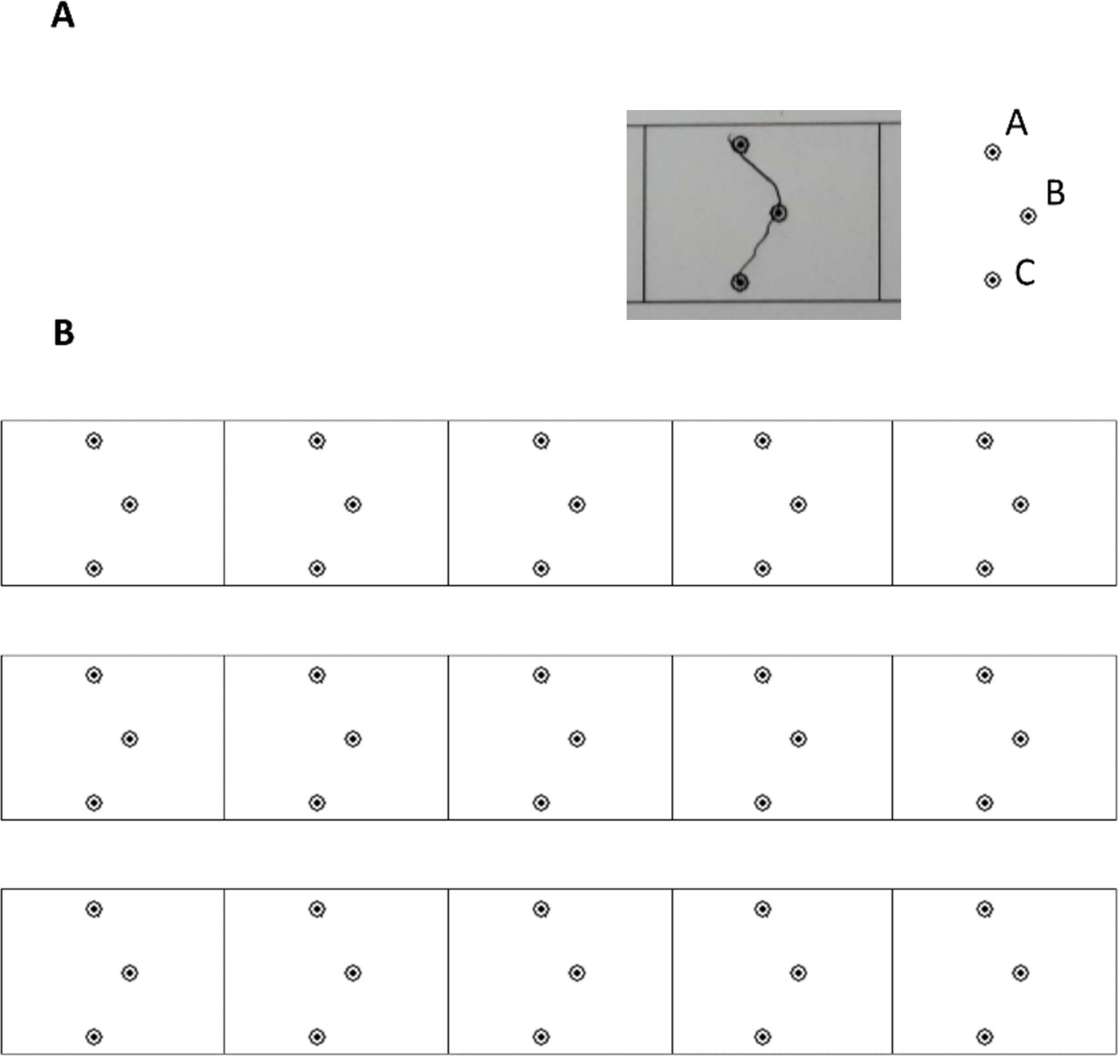
The invented letter stimuli. (A) A single stimulus. Writing direction A-B-C. (B) A block of the invented letter task. Writing direction is from right-to-left.

The ILT [33] was used to study the time-dependent course of motor skill acquisition. The task consists of point-to-point planar movements (Fig 1A: A goes to B goes to C, segment length 1.2 cm, circle outer diameter 3 mm, shape width 6 mm) to form an invented letter. Movement progression within a block (Fig 1B) was from right-to-left (as in Hebrew writing). Participants performed the writing-like task using an HB pencil. Overall, 20 blocks of the task were performed; 12 on the first (training) day, four blocks at 2- or 4-hours post-training, and an additional four blocks on the 2 weeks post-training day. Each block contained 15 repetitions of the same pattern (i.e., repeatedly connecting the point-to-point pattern in Fig 1A). Blocks were separated by 15–30 seconds [8]. Before training and before each test, participants practiced 4 rows of the task. This amount was found to suffice a 5-year-old and was set to ensure equal practice in children and adults. Participants were instructed to connect the dots as quickly and accurately as possible, continuously in one segment, and without making corrections.

### Procedure

On Day 1, children and adults performed 12 blocks of the ILT task. Two hours post-training, 21 children and 20 adults performed 4 additional blocks. Four hours post-training, the remaining 24 children and 20 adults performed the additional 4 blocks. Children and adults were retested for 4 additional blocks two weeks post-training. Children and adults did not practice the task outside the sessions with the experimenter. In the post-training delay interval, children and adults resumed their activities (children in the kindergarten, and adults at the university).

#### Dependent measures

Learning was evaluated using two measures: performance-time and accuracy. Performance times were recorded using a stopwatch, from initial to end-of-writing on a block page (Fig 1B). Accuracy was coded as the number of correctly produced shapes. Erroneous shapes included shapes that were *not* produced in one continuous movement (e.g., a shape that was composed of two segments) or shapes that were too narrow or too wide with respect to the midpoint of the shape (when the line did not go through the encircled Point B in Fig 1A). The 20 task blocks were coded into 5 time-points, each representing 4 blocks. It should be noted that performance-time and accuracy in motor skill learning tasks are considered to reflect two processes that, at least partially, are not overlapping [36]. Accuracy is more related to explicit attentional processes, specifically in children [15, 26].

## Results

**Fig 2:**
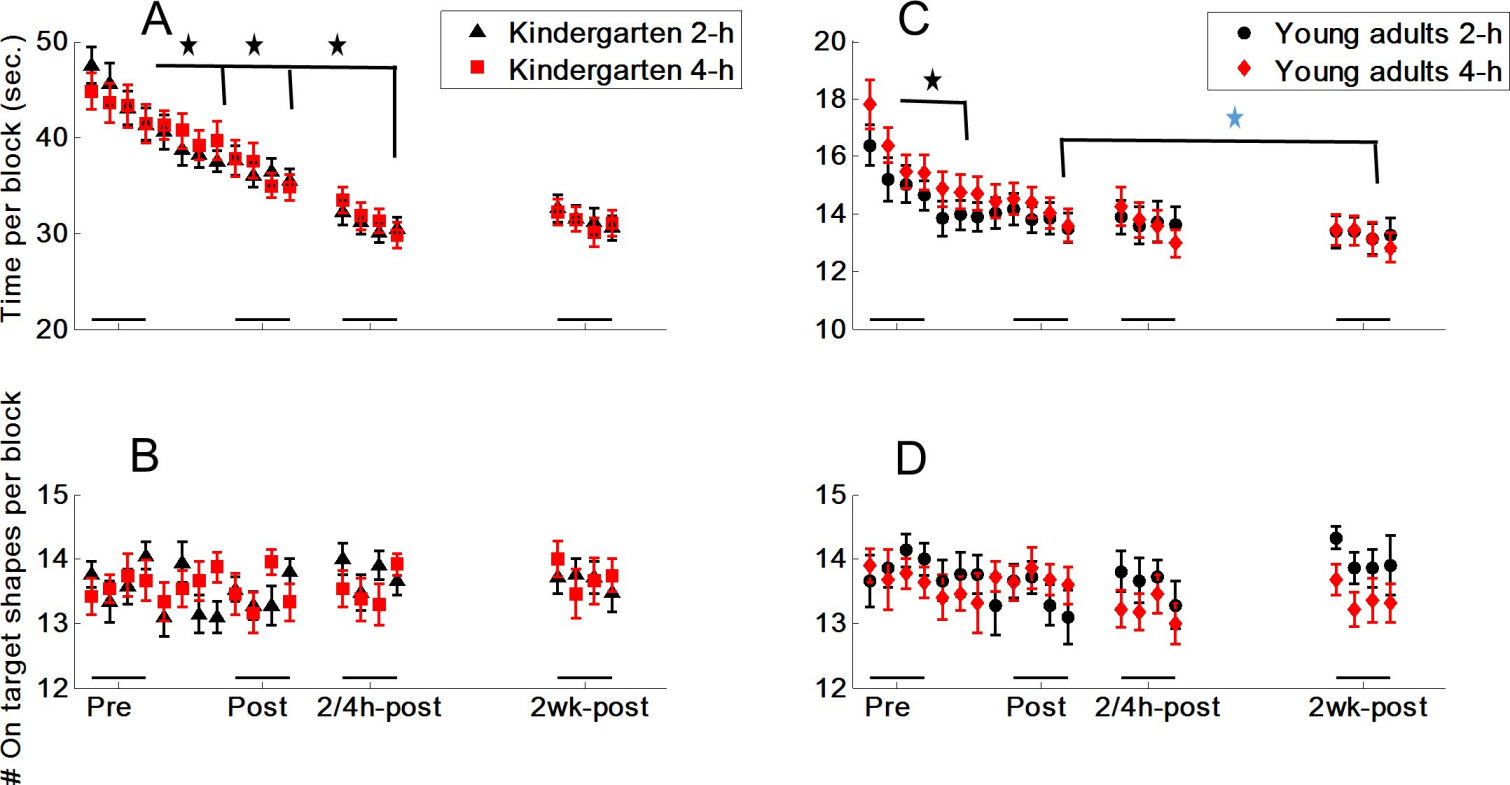
Speed and accuracy (mean and SE): Initial-training (pre, blocks 1–4 on Day 1), end-of-training (post, blocks 9–12 on Day 1), 2- or 4- hours post-training (2/4 h-post), and 2 weeks post-training (2wk-post). (A) Time per block. (B) Number of correctly produced shapes (of 15). Bonferroni, black star, p < .001, blue star, p < .02.

Performance-time and accuracy data are displayed in Fig 2. Raw data analysis of performance times is included to show *within* age-group learning, which was tested using a 5 (time-point = initial-training, mid-training, end-of-training, 2- or 4-hours post-training, two weeks post-training) x 2 (delay-interval of 2- or 4-hours) repeated measure analysis of variance (rm-ANOVA).

The focus of the current study is on the timing of the between-session enhancement (gains) *within* each age-group. Learning of the ILT was shown to involve between-session enhancement in both children and adults [8]. Nevertheless, it also is of interest to test whether the within age-group timing of enhancement significantly differs between age-groups. Groups with different baseline performance times are difficult to compare. For this reason, we included analyses of normalized data [37]. For each participant, performance-time data across all blocks was Z transformed. A 5 (time-point) x 2 (delay-interval) x 2 (age-group = child/adult) rm-ANOVA was applied to Z-transformed performance-times. Accuracy in this task was high in both groups, and was therefore subjected to the full rm-ANOVA on raw data. Because we specifically focus on age-differences in the timing of the between-session enhancement, differences in the gains of the between-session intervals were analyzed separately.

### Raw performance times within age-groups

#### 5-year-olds

The analysis indicated that performance times decreased across the 5 time-points, F(4, 172) = 131.77, p < .001, η_p_^2^ = .75. Post-hoc analysis indicated significant improvement in performance times across the training (from beginning- to mid- to-end-of-training) and from the end-of-training to the test at the 2/4 h delay (Bonferroni's, p < .001). As can be seen in Fig 2, there was no improvement from the delay interval to 2-weeks post-training. There was no delay-interval (2/4 hours post-training) main effect or interaction, indicating that performance was similar across the two delay intervals.

Children's improvement post-training may have stemmed from fatigue accumulated during training, especially towards the end (although there was a break between blocks), which may have lowered their level of performance. To test this possibility, children's performance on the last 4 blocks of the training was compared with an extrapolation of a power-law function of the form “a*x^-b^ + c” fitted to the first 8 training blocks to 4 additional blocks [34, 38]. The findings indicate that actual performance was as expected in Blocks 9 and 10, however performance on the last two blocks of the training session was actually better than expected (by 2.12 and 2.38 sec.; t(44) = 2.24, 2.63, p < .03,.02 for Block 11 and 12, respectively), indicating no effects of fatigue.

#### Adults

The analysis indicated that performance times decreased across the 5 time-points, F(4, 152) = 34.74, p < .001, η_p_^2^ = .48. Post-hoc analysis indicated significant improvement in performance times from initial-training to mid-training (Bonferroni, p < .001), and from mid-training to 2 weeks post-training (Bonferroni, p < .01). Two-week post-training performance was also significantly better than end-of-training performance (Bonferroni, p < .02). There was no delay-interval main effect or interaction, indicating that performance was similar across the two delay intervals (2/4 hours post-training).

It should be noted that in adults, we did not see the improvement above a power-law fitted to the first eight training blocks, and performance was at the level expected by the extrapolation, indicating no effect of fatigue in adults as well.

### Z-transformed performance times across groups

The analysis above suggests that learning enhancement in children is already expressed at 2 hours post-training—earlier than in adults. In adults, the process is more prolonged, and post-practice enhancement is seen only from the delay interval to 2 weeks post-training. However, due to the difference in baseline performance between children and adults, it is difficult to compare their relative improvements. The analysis of the Z-transformed data (Table 1) tested whether there is a difference in the rate of improvement between the groups across the 5 time-points. Because data are normalized, no overall age-group differences were expected to appear.

**Table 1:** Z-transformed data analysis.

	F overall	F training	F 2/4 hours	F retention
Time-point	176.17***	136.33***	57.36***	1.78
Time-point x Age-group	9.39***	2.94	17.64***	3.52‡
Time-point x Delay-group	0.85	1.09	.03	0.15
Time-point x Age-group x Delay-group	1.72	5.05	.48	0.83

***p < .01

‡*p* = .06.

Note. Because data were individually Z-transformed, there are no overall group main effects. Training: comparison between the initial and the last four training blocks; 2/4 hours: comparison between performance at last four training blocks and the four blocks following the delay interval; Retention: comparison between performance following the 2/4-hour delay interval and 2 weeks post-training.

The analysis of the Z-transformed performance times indicated that performance times decreased across the 5 time-points, F(4, 324) = 176.17, p < .001, η_p_^2^ = .69. There were no delay-interval main effects or interactions, indicating that performance was similar across the two delay groups within each age-group. The findings, however, indicated a time-point x age-group interaction, F(1, 81) = 9.39, p < .001, η_p_^2^ = .10. The interaction emerged due to the larger gains attained by children across the 2/4 hours delay-interval, F(1, 81) = 17.64, p < .001, η_p_^2^ = .18, whereby only children significantly improved their performance (F(1,43) = 141.67, p < .001, η_p_^2^ = .77). Furthermore, a marginal interaction emerged from the delay-interval to the 2-week post-training testing, F(1, 81) = 3.52, p = .06, η_p_^2^ = .04, whereby only adults, marginally, improved their performance, F(1,38) = 3.87, p = .06, η_p_^2^ = .09.

### Analysis of accuracy data

The 5 (time-point) x 2 (delay-interval = 2/4 hours) x 2 (age-group = child/adult) rm-ANOVA applied to accuracy data did not indicate any main effects or interactions. Furthermore, no effects emerged when the 5 (time-point) x 2 (delay-interval = 2/4 hours) rm-ANOVA was studied within age-groups. Overall, accuracy was high in both children and young adults.

### Analyses of the differences in performance times and accuracy between blocks

Another possibility for overcoming baseline performance differences between children and adults in testing between-session (off-line) learning enhancement is to analyze the between-session intervals (1^st^ between-session interval: end-of-training to 2/4 hours post-training; 2^nd^ between-session interval: 2/4 hours post-training to retention testing) [14] within age-groups, and to compare differences between age-groups. Here, we report the results both for the raw data as well as for the Z-transformed data.

The 2 (between-session intervals) x 2 (delay-interval = 2/4 hours) x 2 (age-group = child/adult) rm-ANOVA applied to the performance-time raw data indicated a between-session interval main effect, F(1,81) = 43.11, p < .001, η_p_^2^ = .35, an age-group main effect, F(1,81) = 38.74, p < .001, η_p_^2^ = .32, and a significant between-session interval x age-group interaction, F(1,81) = 43.25, p < .001, η_p_^2^ = .35. The interaction emerged because children gained more in the first between-session interval (2/4 h post training) than in the second between-session interval (at two weeks post-training), t(44) = 7.49, p < .001, while no difference emerged in adults.

A similar between-session interval x age-group interaction emerged for the Z-transformed data (the analysis indicated a between-session main effect, F(1,81) = 17.74, p < .001, η_p_^2^ = .18; an age-group main effect, F(1,81) = 6.84, p = .01, η_p_^2^ = .08; and a significant interaction between these factors, F(1,81) = 14.06, p < .001, η_p_^2^ = .15). In children, these data indicated a significantly larger enhancement in the first interval than in the second, t(44) = 7.97, p < .001, while no difference emerged in adults.

The analysis of accuracy data did not indicate any significant main effects or interactions.

### Within age-group associations

Previous studies have shown that lower initial performance on the ILT is associated with higher training gains within age-groups. However, initial performance was not associated with 24 h post-training gains [8]. In the current study, lower initial speed is associated with higher training gains as well (5-year-olds, r(45) = -.68, p < .001; adults, r(40) = -.63, p < .001). The association stems from an association between lower initial performance and the larger gains attained from initial- to mid-training (5-year-olds, r(45) = -.76, p < .001; adults, r(40) = -.68, p < .001), and *not* from the smaller gains attained from mid-training to its end. In children the association extends beyond training, and lower initial performance was associated with larger post-training delay gains (r(45) = -.41, p < .01, significantly different than the association in adults, (r(40) = .07, n.s.; Z = 2.25, p < .05). As in previous studies [19], early enhancement in children’s performance times was correlated with lower end-of-training performance, r(45) = -.47, p < .001. Correlations between initial or end-of-training performance and later gains (i.e., from delay to 2 weeks post-training) are insignificant. Furthermore, training gains and early/late post-training gains were not associated in any of the age-groups.

## Discussion

The findings of the current study reveal that 5-year-old children show performance enhancement of a newly learned motor skill as early as a few hours post-training, while in adults such enhancement takes longer to develop [14, 15, 17]. Children's early enhancement was retained two weeks later, suggesting that the processes that enabled the enhancement had stabilized. In both groups, slower initial performance was associated with larger training gains, but in children, it was also associated with larger early enhancement, suggesting short-term associations. In both age-groups however, the later between-session performance change (from 2/4 h post-training to 2 weeks post-training) was not associated with initial or end-of-training performance, suggesting that this 2-week change reflects long-term processes.

Children and adults improved similarly during the ILT training. However, they showed different patterns at re-testing. While improvement in children occurred a few hours post-training and was retained 2 weeks later, it was a more slowly occurring process in adults, with significant improvement achieved only at the 2 weeks post-training testing vs. end-of-training performance level. Children had larger gains in performance times in the early between-session interval (few hours post training, i.e. from end-of-training to 2/4 hours post-training) than in the later between-session interval (from 2/4 hours post-training to 2 weeks post-training), while in adults these gains were of a similar magnitude. As indicated by the analysis of the Z-transformed data, relatively larger gains were found in children than in adults in the early between-session enhancement while the opposite occurred for the later between-session interval. These data further stress developmental differences in motor skill learning processes. In a previous study using the same task [8], significant gains in children and adults, assessed at 24 h post-training, were retained 2 weeks post-training. This suggests that although at 2 or 4 hours after training adults do not develop performance enhancement, such an enhancement is expressed a day later.

In both age-groups, lower initial performance was associated with higher training gains attained during the training, particularly during the initial part of the training. However, only in children did this association extend beyond training, whereby lower initial performance was associated with higher post-training early gains attained a few hours post-training. Twenty-four hours post-training enhancement is not related to initial skill level [8], suggesting that at least partially different neuronal processes subserve a few-hours post-training and 24 hours post-training performance enhancements in children. This association suggests that unlike later enhancements, which are more related to long-term memory processes, the early enhancement may be related to processes that enhance performance during training [15, 19], and may reflect neuronal processes subserving the experience-driven modification of the motor system [20, 39–41].

Different neuronal processes may underlie synapse plasticity in children versus adults, and in early formation vs. later stabilization processes [24, 42, 43]. These differences may explain the developmental differences in the timing of between-session performance enhancement outlined here. Recent animal studies have found rapid formation of new dendritic spines after just a few minutes of training [22]. Dendrites play a critical role in integrating synaptic inputs and in determining the extent to which action potentials are produced by the neuron. Synaptic input cluster formation in juvenile barn owls appears to require the production of new synapses, while cluster formation in adults does not, possibly using sleep-dependent remodeling mechanisms [23, 42]. The current study adds to the growing literature on earlier expression of delayed gains in speed of performance in children, in motor learning tasks [44, 14–16], and in adaptation tasks [13]. A childhood advantage in recall after a one-hour delay of word forms was reported as well [45]. These findings suggest that spacing within learning protocols may show an age-dependent effect. Furthermore, these findings have implications for school instructional schedules. The findings of the current study suggest that consolidation is achieved earlier in young children [14]. These findings support the notion that in primary school, lessons of the same subject can be distributed across a school day, with appropriate breaks in between (e.g., one math lesson in the morning and a second two hours later). For adolescents, lessons of the same subject should be scheduled on different days to allow for appropriate consolidation processes to take place. Of course, these findings should be tested in large, varied samples.

In the current study, we did not find any difference between the 2 h and 4 h delay groups on the ILT in both children and adults [14], suggesting that this time interval does not differentiate post-training performance, at least not in this paradigm [46], although longer delays may have a differential effect [47]. It should be noted that a performance boost in young adults 5–30 minutes post-training that decays at 4 hours post-training was reported for different paradigms [48–50]. However, no studies tested 2 hours post-training, and the findings of the current study suggest that that the previously observed boost may already decay at 2 hours post-training.

There exist several standardized tests of declarative memory for preschool children (e.g., within the Kaufman Assessment Battery for Children, K-ABC-II [51] and the children’s memory scale [52, 53]. However, there are no standardized tests that assess procedural memory. One reason may be that available tasks for testing skill learning are too difficult for young children. The ILT used in the current study was designed for kindergarten children. The task has low accuracy demands, which enable improvement in speed while maintaining high accuracy scores [8, 33]. This differentiates the ILT from other tasks commonly used for assessing motor skill learning, such as the finger-tapping task (FTT, [54]), the SRTT [55], or the mirror drawing task [30]. When adult versions of these task are given to pre-primary children, the children achieve low accuracy scores, with accuracy demands possibly affecting the speed of performance [15, 17, 25, 56]. Another reason may be that it is difficult to assess long-term memory 24 h post initial learning. The finding that enhancement (or its absence) can be assessed shortly after practice impacts future studies of skill learning in different child samples [33, 34]. This raises the option of using long-term memory measures of tasks as the ILT as a predictor of academic learning [29], or other developmental outcomes.

The conclusions of this study must be considered within its limitations. The current grapho-motor task was used for studying developmental differences in the timing of memory enhancement. Only one age-group of children was tested; these children were 5-year-olds who have not yet mastered penmanship. Likewise, only one age-group of adults was tested. A variety of age groups may have revealed different findings, although a similar study in somewhat older children revealed similar findings [14]. The difference in penmanship between the age-groups was related to performance differences (time and accuracy), but it is less likely that it affected the timing of between-session enhancement. An option might have been to use a single performance measure (combining both speed and accuracy [57]) in tasks in which these measures reflect shared processes. Although this may hold true in general, it is not clear if this is the case in children, where accuracy is much more related to attention. Future studies should include larger samples and a more varied pool of initial assessments, as well as learning and memory tasks that are similarly novel to both children and adults, to enable a broader view of the findings.

## Abbreviations

ILT: Invented Letter Task
